# Incidence of hospital-acquired acute kidney injury and trajectories of glomerular filtration rate in older adults

**DOI:** 10.1186/s12882-023-03272-5

**Published:** 2023-08-01

**Authors:** Natalie Ebert, Alice Schneider, Doerte Huscher, Nina Mielke, Yanina Balabanova, Gunnar Brobert, Carla Lakenbrink, Martin Kuhlmann, Anne-Katrin Fietz, Markus van der Giet, Volker Wenning, Elke Schaeffner

**Affiliations:** 1 Charité-Universitätsmedizin Berlin, Institute of Public Health, Berlin, Germany; 2 Institute of Biometry and Clinical Epidemiology, Charité-Universitätsmedizin Berlin, Institute of Public Health, Berlin, Germany; 3 Bayer AG, Berlin, Germany; 4 Department of Nephrology, Vivantes Klinikum im Friedrichshain, Berlin, Germany; 5 Division of Nephrology and Intensive Care, Charité-Universitätsmedizin, Berlin, Germany; 6 AOK Nordost - Die Gesundheitskasse Berlin, Berlin, Germany

**Keywords:** Acute kidney injury (AKI), Hospital-aquired AKI, AKI incidence, Kidney function, In-hospital complication, eGFR trajectories, Older adults

## Abstract

**Background:**

In older adults, epidemiological data on incidence rates (IR) of hospital-acquired acute kidney injury (AKI) are scarce. Also, little is known about trajectories of kidney function before hospitalization with AKI.

**Methods:**

We used data from biennial face-to-face study visits from the prospective Berlin Initiative Study (BIS) including community-dwelling participants aged 70+ with repeat estimated glomerular filtration rate (eGFR) based on serum creatinine and cystatin C. Primary outcome was first incident of hospital-acquired AKI assessed through linked insurance claims data. In a nested case-control study, kidney function decline prior to hospitalization with and without AKI was investigated using eGFR trajectories estimated with mixed-effects models adjusted for traditional cardiovascular comorbidities.

**Results:**

Out of 2020 study participants (52.9% women; mean age 80.4 years) without prior AKI, 383 developed a first incident AKI, 1518 were hospitalized without AKI, and 119 were never hospitalized during a median follow-up of 8.8 years. IR per 1000 person years for hospital-acquired AKI was 26.8 (95% confidence interval (CI): 24.1–29.6); higher for men than women (33.9 (29.5–38.7) vs. 21.2 (18.1–24.6)). IR (CI) were lowest for persons aged 70–75 (13.1; 10.0-16.8) and highest for ≥ 90 years (54.6; 40.0-72.9). eGFR trajectories declined more steeply in men and women with AKI compared to men and women without AKI years before hospitalization. These differences in eGFR trajectories remained after adjustment for traditional comorbidities.

**Conclusion:**

AKI is a frequent in-hospital complication in individuals aged 70 + showing a striking increase of IR with age. eGFR decline was steeper in elderly patients with AKI compared to elderly patients without AKI years prior to hospitalization emphasising the need for long-term kidney function monitoring pre-admission to improve risk stratification.

**Supplementary Information:**

The online version contains supplementary material available at 10.1186/s12882-023-03272-5.

## Background

Acute kidney injury (AKI) is a common in-hospital complication. In the general population, the reported incidence of hospital-acquired AKI varies between seven and 18% depending on the burden of disease and age of the studied population [1]. Results from a large multicenter cross-sectional study show that AKI occurred in more than half of patients on intensive care unit [2]. There is strong evidence that AKI is associated with increased risk of cardiovascular (CV) events and death, prolonged hospitalization, progression of chronic kidney disease (CKD) [3] and end-stage kidney disease all of which result in a large societal burden [4–7]. AKI also imposes a financial burden for health insurances and health care systems due to longer hospitalizations, increased utilization of intensive care and kidney replacement therapy [8, 9]. The incidence of AKI is particularly high in older adults representing a common complication among hospitalized patient. Thus epidemiologic data assessed in a community-dwelling older population are indispensable to gain a better understanding of AKI risk and to reduce the AKI burden in geriatric inpatients.

When investigating kidney function decline in the context of AKI most studies model kidney function trajectories after patients developed in-hospital AKI to show recovery or worsening [10, 11]. However, little is known about kidney function decline in older patients before hospitalization with AKI. Trajectories of estimated glomerular filtration rate (eGFR) have been acknowledged as a more valid and informative measure of kidney function compared to single estimates [12]. Also, changes of eGFR over time have been evaluated as a surrogate end point for progression of CKD [13]. Characterizing long-term changes in eGFR prior to hospitalization could improve risk stratification for identifying older patients most at risk for developing AKI.

Using longitudinal data from the population-based Berlin Initiative Study (BIS) we investigate first incidence of hospital-acquired AKI with a particular focus on old and very old adults (stratified by age, sex and pre-existing comorbidities). We hypothesize that patients aged 70 years and older with AKI have a steeper decline of eGFR already prior to hospitalization compared to patients aged 70 + hospitalized without AKI independent of traditional CV comorbidities.

## Materials and methods

### Study population and data collection

The Berlin Initiative Study (BIS) is a community-dwelling cohort study of 2069 adults aged 70+ years in Berlin, Germany. A detailed description of the BIS can be found elsewhere [14, 15]. In brief, all participants are members of one of the largest German statutory health insurance (AOK Nordost – Die Gesundheitskasse). For detailed in- and exclusion criteria see Supplemental Methods. Enrolment (baseline visit) took place from November 2009 to June 2011. After baseline, four follow up visits were conducted biennially and the dataset was closed on November 30, 2019. Study visits included standardized face-to-face interviews assessing demographics, lifestyle variables, medications and comorbidities. Anthropometric variables were measured and blood and urine samples were collected. At all study visits kidney function was assessed with serum creatinine using an isotope-dilution mass spectrometry-traceable enzymatic method and cystatin C measured with standardized particle-enhanced nephelometry [15]. The clinical information obtained during interviews was linked to insurance claims data from the AOK Nordost. In addition, claims data were available retrospectively 3–4 years prior to study start. The study was approved by the ethics committee of the Charité University Hospital Berlin, Germany (EA2/009/08). The BIS adheres to the Declaration of Helsinki and rules of “Good Scientific Practice”. All study participants gave written informed consent.

### Exclusion criteria

Participants were excluded if their insurance status was invalid or if a prior diagnosis of AKI defined with the ICD-10 code N17.xx was present in the claims data within three years before study entry.

### Outcome variables

We assessed first incident hospital-aquired AKI in a population of community-dwelling older adults. The analysis was restricted to in-hospital AKI defined based on insurance claims data (ICD-10: N17.xx), excluding patients who were admitted to hospital with the diagnosis of AKI. Since the exact date of AKI was not available we defined the day of hospital admission as event date. Study cohort members were followed from study cohort entry until 30.11.2019 (dataset closure) or death, whatever occurred first.

Secondly, we restricted the analysis to patients with hospitalizations and modeled eGFR trajectories based on multiple eGFR measurements assessed during the biennial study visits. We chose a nested case-control design to identify two groups of study participants: those who were hospitalized and developed AKI (cases) and those hospitalized without AKI (controls). For modelling eGFR trajectories we included all eGFR estimates assessed during the study visits before hospital admission. The focus of this analysis was to compare eGFR trajectories prior to hospitalization with or without AKI event. Thus the anchor for the analysis was the hospitalization event. GFR trajectories were modeled based on date of eGFR measurement. eGFR was calculated with the BIS2 equation [15]. Covariates were selected into the model based on knowledge and published data [16].

### Covariates for modelling eGFR trajectories

All covariates were included as time-dependent for eGFR trajectories: age, UACR, CRP, diabetes mellitus, arterial hypertension, smoking, polymedication, body mass index (BMI), and number of prior hospitalizations, blood pressure; they were included at baseline and reassessed at each study visit.

The following covariates were defined as “chronic disease” based on data assessed at study inclusion and new incident cases were included during follow-up (self-reported or ICD-10 based) until dataset closure or death: peripheral artery disease (PAD), congestive heart failure (CHF), atrial fibrillation (AF), prior stroke and prior myocardial infarction (MI). Further covariate definitions are described in the Supplemental Methods (*covariates*).

### Verification of ICD-10-based AKI diagnosis using a chart review

To verify the validity of the ICD-10-based AKI diagnosis we performed a chart review including all participants with the N17.xx diagnosis in the claims data during the observation period. At each study visit, participants were asked to provide their hospital reports including laboratory results. Thus, based on the information available, hospital reports were singled-out and all information on AKI diagnosis and lab results during hospitalization was collected to verify the claims-data based diagnosis of AKI (increase in serum creatinine of ≥ 0.3 mg/dl within 48 h during hospitalization). For the verification process of claims data-based AKI definition we also used data from recurrent AKIs. This is in contrast to the main study where recurrent AKIs were not included into the analysis.

### Statistical analysis

Descriptive analysis includes means with standard deviation (SD) or medians with interquartile range (IQR) for continuous variables, and absolute frequencies with percentages for categorical variables. Incidence rates (IR) of first incident hospital-acquired AKI were calculated per 1000 person years, with 95% confidence intervals (CI) assuming that the observed number of cases follows a Poisson distribution. IR were described by sex, age stratum and pre-existing traditional comorbidities at baseline.

In a nested case-control study we compared eGFR trajectories in patients with AKI during the years before hospitalization with eGFR trajectories in patients without AKI. To identify cases (patients hospitalized with AKI) and controls (patients hospitalized without AKI) we performed a matched-pair analysis and chose age at the study visit preceding hospitalization (continuous), sex (male/female) and months between the study visit preceding hospitalization and day of admission (maximum difference between matched pairs: three months) as matching criteria. For the AKI group we used the first hospitalizations with AKI as an “anchor”, while for the controls the hospitalization with the optimal match with regard to matching criteria was chosen, which was not necessarily the first hospitalization. Thus, we selected two equally large groups of patients with and without AKI to model eGFR trajectories prior to hospital admission.

eGFR trajectories were calculated using linear mixed-effects models with clustering of subjects for longitudinal data (random intercept model), based on repeat eGFR values of biennial study visits. This approach allows to include patients with single eGFR values with the aim to reduce selection bias under the missing-at-random assumption [17, 18]. eGFR trajectories were modeled based on the date of each eGFR measurement. We considered time, AKI, and the interaction of time and AKI as covariates of interest and included a priori selected covariates (see description below). Due to expected sex-specific differences we estimated the models for males and females separately [16]. Age was included both as linear and quadratic term and was centered for modelling; time was modeled as a natural cubic spline with 3 degrees of freedom. We calculated the eGFR trajectories using three models: one including age as the only variable, a second model with age, log-transformed UACR (continuous), diabetes mellitus, arterial hypertension, CHF, PAD, MI, stroke, and AF, and a final model, where in addition to the second model we included BMI, smoking, polymedication (≥ 5 medication), number of prior hospitalizations, and log-transformed CRP (continuous). Medications were not included as they were considered parts of the causal pathway. We modeled eGFR trajectories for graphical illustration using all eGFR values of study participants based on averaged prevalence of the respective comorbidities, consequently all categorical variables (0 = no; 1 = yes) were included as continuous variables into the models (i.e. a prevalence of 25% resulted in a value of 0.25). We accounted for missing data by using multiple imputation by chained equation [19]. All covariates of the models were included to generate ten imputed datasets. We used predictive mean matching for continuous variables [20]. eGFR values can be missing due to omitted visits or refusal/mispuncture during blood sampling.

All analyses were carried out using IBM SPSS Statistics version 26 and 27, and R version 3.6.1 applying packages mice, lme4, splines, ggeffects, epitools (function pois.exact) and forestplot and reporting of results was performed according to the Strengthening the Reporting of Observational Studies in Epidemiology (STROBE) statement (Supplement).

## Results

### Baseline characteristics of hospitalized individuals

Out of 2069 participants of the BIS, 12 individuals were excluded due to an invalid health insurance status during follow-up and 37 individuals due to a documented AKI diagnosis prior to study inclusion resulting in a final study sample of 2020 individuals (Figure S1). During a median observation time (IQR) of 8.8 (5.9–9.3) years, 383 individuals developed a first incident hospital-acquired AKI, 1518 were hospitalized without AKI, and 119 were never hospitalized. Table 1 shows the baseline characteristics of hospitalized individuals (n = 1901) for those with and without AKI at cohort entry.

**Table 1:** Baseline characteristics of all hospitalized study participants stratified by AKI status

	Total n = 1901 (10%)	With AKI n = 383 (20%)	Without AKI n = 1518 (80%)
Age, years (mean, ± SD)	80.5 ± 6.6	82.2 ± 6.3	80.1 ± 6.6
70–74	497 (26.1)	61 (15.9)	436 (28.7)
75–79	447 (23.5)	78 (20.4)	369 (24.3)
80–84	403 (21.2)	103 (26.9)	300 (19.8)
85–89	364 (19.2)	95 (24.8)	269 (17.7)
≥ 90	190 (10.0)	46 (12.0)	144 (9.5)
Female	1004 (52.8)	170 (44.4)	834 (54.9)
Number of medications	4.9 ± 3.0	6.1 ± 3.1	4.6 ± 2.9
Smoking			
never	962 (50.6)	169 (44.1)	793 (52.2)
past	836 (44.0)	194 (50.7)	642 (42.3)
current	101 (5.3)	20 (5.2)	81 (5.3)
Physical activity (> 30 min)			
< 1x /week	486 (25.6)	124 (32.4)	362 (23.8)
1-5x /week	880 (46.3)	157 (41.0)	723 (47.6)
> 5x /week	530 (27.9)	100 (26.1)	430 (28.3)
Systolic blood pressure, mmHg	145.4 ± 21.9	146.4 ± 22.4	145.2 ± 21.8
Diastolic blood pressure, mmHg	81.3 ± 13.1	80.1 ± 13.4	81.6 ± 13.0
BMI, kg/m²	27.7 ± 4.2	28.4 ± 4.5	27.6 ± 4.2
BMI < 30, kg/m²	1394 (73.3)	257 (67.1)	1137 (74.9)
BMI ≥ 30, kg/m²	506 (26.6)	125 (32.6)	381 (25.1)
Creatinine, mg/dl	1.0 ± 0.3	1.2 ± 0.4	1.0 ± 0.3
Cystatin C, mg/l	1.2 ± 0.4	1.3 ± 0.4	1.1 ± 0.3
Albumin, g/l	39.9 ± 3.1	39.5 ± 3.1	40.0 ± 3.0
CRP, mg/l	1.8 [0.9; 3.6]	2.4 [1.2; 4.8]	1.7 [0.9; 3.3]
HbA1c, %	6.1 [5.8; 6.4]	6.2 [5.8; 6.5]	6.0 [5.8; 6.4]
Urea, mg/dl	41.7 [34.5; 51.7]	47.2 [38.8; 9.4]	40.4 [33.9; 49.6]
eGFRBIS2, ml/min/1.73 m²	57.9 ± 15.1	50.5 ± 14.4	59.8 ± 14.6
eGFRBIS2 ≥ 60 ml/min/1.73 m²	878 (46.2)	95 (24.8)	783 (51.6)
eGFRBIS2 < 60 ml/min/1.73 m²	1022 (53.8)	288 (75.2)	734 (48.4)
UACR, mg/g	11.1 [4.6; 30.6]	17.6 [6.6; 71.6]	9.6 [4.3; 27.0]
UACR < 30 mg/g	1398 (73.5)	229 (59.8)	1169 (77.0)
UACR ≥ 30 mg/g	487 (25.6)	151 (39.4)	336 (22.1)
Diabetes mellitus	500 (26.3)	138 (36.0)	362 (23.8)
Arterial hypertension	1520 (80.0)	342 (89.3)	1178 (77.6)
Congestive heart failure	501 (26.4)	136 (35.5)	365 (24.0)
Atrial fibrillation	286 (15.4)	85 (22.2)	201 (13.2)
Peripheral artery disease	119 (6.3)	39 (10.2)	80 (5.3)
Stroke	209 (11.0)	55 (14.4)	154 (10.1)
Myocardial infarction	287 (15.1)	92 (24.0)	195 (12.8)
Anemia^a^, n (%)	331 (17.4)	94 (24.5)	237 (15.6)
Cancer	524 (27.6)	112 (29.2)	412 (27.1)

**Legend**: Values for continuous variables given as mean ± standard deviation, or median [interquartile range]; for categorical variables, as number (percentage). ^a^Anemia: haemoglobin in men < 13 g/dl and in women < 12 mg/dl. Missing values for anthropometric or laboratory values do not exceed 3%

Abbreviations: BIS, Berlin Initiative Study; eGFR, estimated glomerular filtration rate by creatinine- and cystatin C–based BIS2 equation; BMI, body mass index; CRP, C-reactive protein; UACR, urinary albumin-creatinine ratio; HbA1c, glycosylated haemoglobin type A1C.

**Fig. 1:**
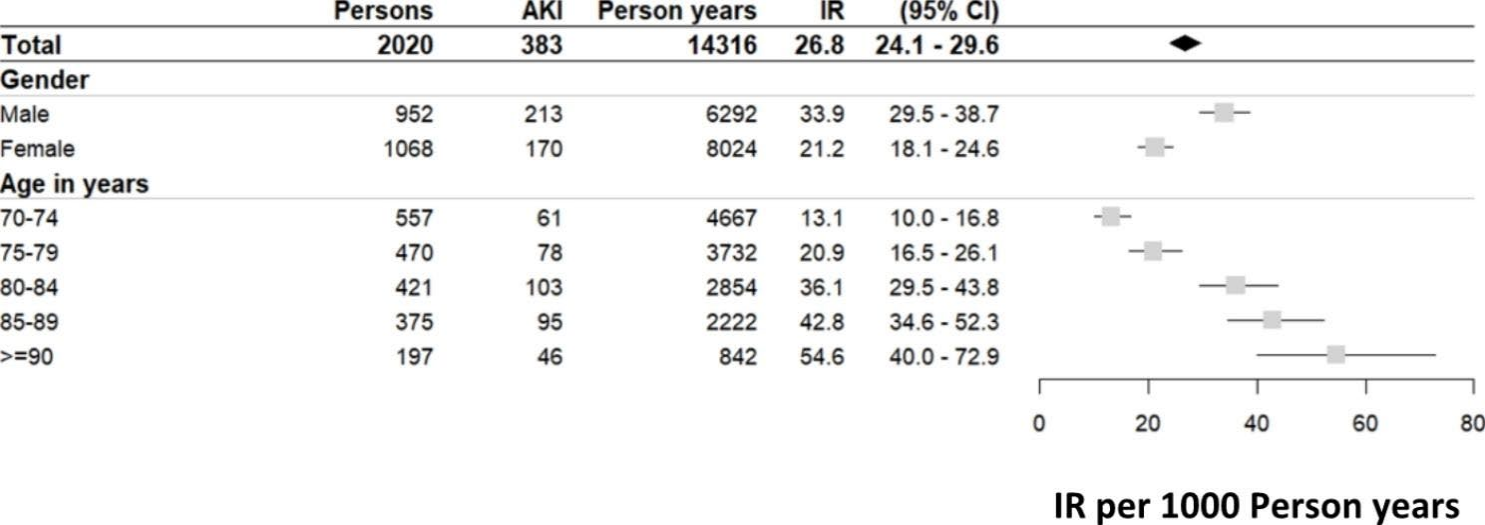
Age and sex stratified incidence rate (per 1000 person years) of AKI in the study population

**Fig. 2:**
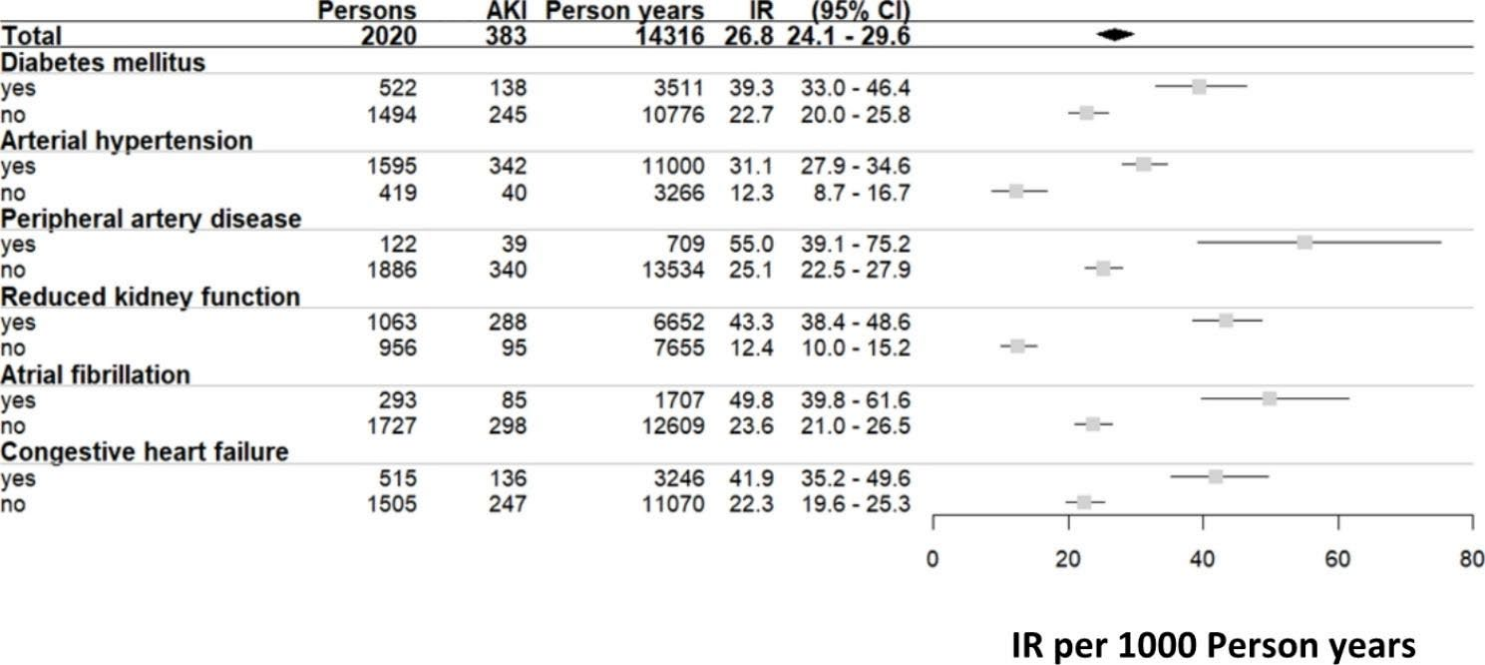
Incidence rates (per 1000 person years) stratified by pre-existing comorbidities *Missing values: 6 values were missing for arterial hypertension, 12 values were missing for peripheral artery disease

At cohort entry, individuals with subsequent hospital-acquired AKI were older (mean age: 82.2 vs. 80.1 years), less likely to be female (44.4% vs. 54.9%), more likely to be a past or current smoker (55.9% vs. 47.6%), to be physically inactive (32.4% vs. 23.8%), to have a BMI of ≥ 30 (32.6% vs. 25.1%), and to be anemic (24.5% vs. 15.6%) compared to those without AKI. Regarding kidney measures, individuals with AKI had higher mean serum creatinine and cystatin C levels (1.2 vs. 1.0 mg/dl and 1.3 vs. 1.1 mg/l, respectively), resulting in a lower mean eGFR_BIS2_ (50.5 vs. 59.8 ml/min/1.73 m²), and had a higher prevalence of UACR ≥ 30 mg/g (39.4% vs. 22.1%). Comorbidities such as diabetes mellitus (36.0% vs. 23.8%), CHF (35.5% vs. 24.0%), AF (22.2% vs. 13.2%), and MI (24.0% vs. 12.8%) were more prevalent in individuals with AKI (Table 1). Baseline characteristics of all 2020 study participants (52.9% women; mean age 80.4 years) show that those who were never hospitalized were younger and overall healthier (Table S1). The total IR per 1000 person years (CI) for first incident AKI was 26.8 (24.1–29.6), higher in men than women (33.9 (29.5–38.7) vs. 21.2 (18.1–24.6)). IR were calculated stratified by age showing the lowest IR in the age category 70–75 years with 13.1 (10.0-16.8) and highest IR in the age category of ≥ 90 with 54.6 (40.0-72.9) (Fig. 1). When comparing IR of AKI in patients with vs. without CV comorbidities we found significantly higher rates in individuals with diabetes mellitus, arterial hypertension, PAD, reduced kidney function, AF, and CHF (Fig. 2). 29 out of 383 (7.6%) individuals with hospital-acquired AKI required dialysis during the hospitalization (data not shown).

### Verification of ICD-10-based AKI diagnosis using a chart review

In 244 out of 569 documented AKI events (43%) hospital reports were available and reviewed for documented N17.xx diagnosis, which could be confirmed in all 244 cases. In 136 of 244 AKI cases (56%) hospital reports included also laboratory test results. According to the latest KDIGO (Kidney Disease Improving Global Outcomes) guidelines the diagnostic criteria for AKI was based on an increase of serum creatinine level of at least 0.3 mg/dl within 48 h [21]. In all 136 cases (24%) with hospital-acquired AKI the diagnosis could be validated with the available laboratory results (Figure S2).

### eGFR trajectories comparing individuals hospitalized with and without AKI

We selected 726 individuals (404 men and 322 women) in a nested case-control study using a matched pairs approach to compare eGFR trajectories from cohort entry until admission to hospital with and without AKI. In the selected sample, mean age was higher (85.1 vs. 80.5 years), less women were included (44.4 vs. 52.6%) and mean eGFR was lower (48.1 vs. 57.9 ml/min/1.73 m²) compared to the 2020 study participants (Tables 1 and 2).

**Table 2:** Characteristics of individuals included in nested case-control study (n = 726) at study visit preceding the hospitalization

	Total (n = 726)	With AKI (n = 363)	Without AKI (n = 363)
Age, years*	85.1 ± 6.5	85.2 ± 6.5	85.1 ± 6.4
70–74	53 (7.3)	24 (6.6)	29 (8.0)
75–79	115 (15.8)	60 (16.5)	55 (15.2)
80–84	185 (25.5)	91 (25.1)	94 (25.9)
85–89	188 (25.9)	96 (26.4)	92 (25.3)
≥ 90	185 (25.5)	92 (25.3)	93 (25.6)
Female*	322 (44.4)	161 (44.4)	161 (44.4)
Time between study visit^a^ and hospitalization (months)*	3.0 [1.4; 4.8]	3.4 [1.6; 5.4]	2.6 [1.2; 4.2]
Length of hospitalization (days)	9 [5; 21]	14 [7; 27]	6 [3; 13]
Number of prior hospitalizations	3 [1; 6]	4 [2; 8]	3 [1; 5]
Number of medications	6.0 ± 3.3	6.7 ± 3.2	5.2 ± 3.1
Smoking			
never	338 (46.6)	158 (43.5)	180 (49.6)
past	348 (47.9)	182 (50.1)	166 (45.7)
current	36 (5.0)	22 (6.1)	14 (3.9)
Physical activity (> 30 min)			
< 1x /week	349 (48.1)	195 (53.7)	154 (42.4)
1-5x /week	259 (35.7)	117 (32.2)	142 (39.1)
> 5x /week	113 (15.6)	49 (13.5)	64 (17.6)
Systolic blood pressure, mmHg	141.4 ± 22.7	141.2 ± 23.3	141.5 ± 22.2
Diastolic blood pressure, mmHg	78.5 ± 13.4	77.8 ± 13.9	79.3 ± 12.9
BMI, kg/m²	27.2 ± 4.6	27.7 ± 4.8	26.6 ± 4.3
BMI < 30, kg/m²	538 (74.1)	252 (69.4)	286 (78.8)
BMI ≥ 30, kg/m²	171 (23.6)	101 (27.8)	70 (19.3)
Creatinine, mg/dl	1.2 ± 0.4	1.3 ± 0.5	1.0 ± (0.3
Cystatin C, mg/l	1.4 ± 0.5	1.6 ± 0.6	1.3 ± 0.4
Albumin, g/l	39.2 ± 3.6	39.0 ± 3.4	39.4 ± 3.8
CRP, mg/l	2.1 [1.0; 4.4]	2.5 [1.2; 5.6]	1.8 [0.9; 3.6]
HbA1c, %	6.0 [5.7; 6.4]	6.0 [5.7; 6.5]	6.0 [5.7; 6.3]
Urea, mg/dl	46.4 [36.6; 59.7]	52.4 [39.8; 66.0]	43.0 [35.0; 52.9]
eGFRBIS2, ml/min/1.73 m²	48.1 ± 14.7	43.0 ± 14.0	53.2 ± 13.7
eGFRBIS2 ≥ 60 ml/min/1.73 m²	148 (20.4)	42 (11.6)	106 (29.2)
eGFRBIS2 < 60 ml/min/1.73 m²	558 (76.9)	310 (85.4)	248 (68.3)
UACR, mg/g	20.0 [6.8; 55.0]	26.9 [9.0; 87.1]	13.8 [5.4; 39.0]
UACR < 30 mg/g	425 (58.5)	184 (50.7)	241 (66.4)
UACR ≥ 30 mg/g	266 (36.6)	158 (43.5)	108 (29.8)
Diabetes mellitus	206 (28.4)	131 (36.1)	75 (20.7)
Arterial hypertension	621 (85.5)	327 (90.1)	293 (80.7)
Congestive heart failure	247 (34.0)	157 (43.3)	90 (24.8)
Atrial fibrillation	243 (33.5)	140 (38.6)	103 (28.4)
Peripheral artery disease	64 (8.8)	44 (12.1)	20 (5.5)
Stroke	138 (19.0)	76 (20.9)	62 (17.1)
Myocardial infarction	199 (27.4)	118 (32.5)	81 (22.3)
Anemia^b^_,_ n (%)	211 (29.1)	117 (32.2)	94 (25.9)
Cancer	197 (27.1)	107 (29.5)	90 (24.8)

**Legend**: Matched-pair analysis was performed to identify cases (with AKI) and controls (without AKI) using the criteria marked with *: age at the study visit preceding hospitalization, sex, months between study visit preceding hospitalization and day of admission)

Values for continuous variables given as mean ± standard deviation or median [interquartile range]; for categorical variables, as number (percentage). ^a^Median study visit of data assessment was 1st follow-up visit (interquartile range: 0 (baseline) to 2^n^d follow up visit) for total, AKI and non-AKI group, respectively. ^b^Anemia: haemoglobin in men < 13 g/dl and in women < 12 mg/dl. Missing values for anthropometric or laboratory values do not exceed 5%

Abbreviations: BIS, Berlin Initiative Study; eGFR, estimated glomerular filtration rate by creatinine- and cystatin C–based BIS2 equation; BMI, body mass index; CRP, C-reactive protein; UACR, urinary albumin-creatinine ratio; HbA1c, glycosylated haemoglobin type A1C.

eGFR trajectories were modeled using all eGFR values assessed during biennial study visits. Of 1867 available visits, 29 eGFR values (1.6%) were missing leaving 1838 eGFR measurements (median (IQR): 2 [1–4] per patient) over a median observation time of 4.4 years. In 26 out of the 726 subjects, at least one eGFR measurement (3.6%) was missing. For 28.8% of participants one eGFR value, for 22.6% two, for 22.2% three, for 19.6% four and for 6.9% five eGFR values were available.

**Fig. 3:**
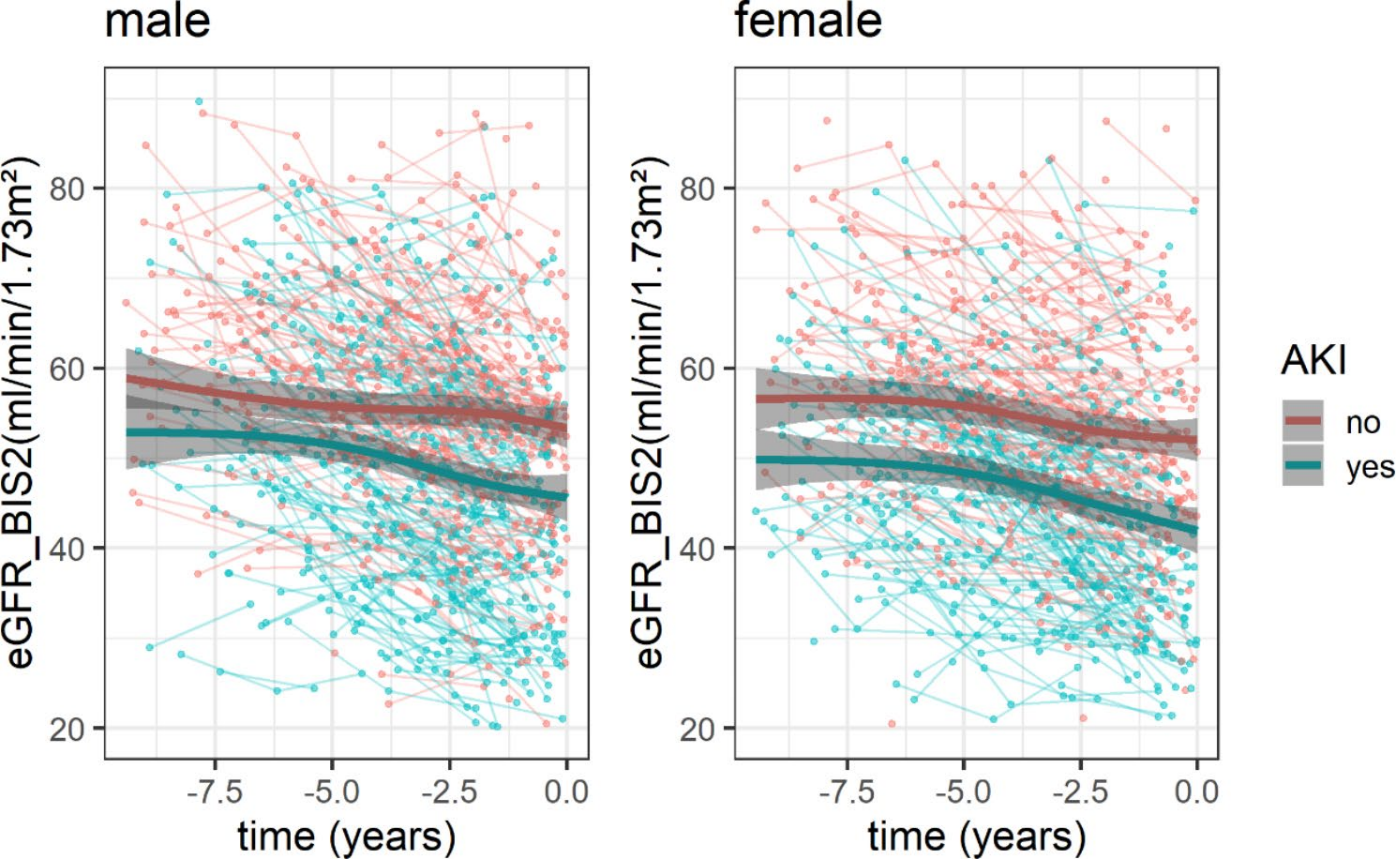
eGFR trajectories of individuals with AKI (green) and without AKI (red) for men and women calculated with a linear mixed-model in a nested case-control study eGFR trajectories were estimated with a mixed-effect model based on pooled results of multiple imputed data with 95% confidence intervals (grey area) for men with (n = 202) and without (n = 202) AKI and women with (n = 161) and without (n = 161) AKI .The x-axis shows the time (years) from inclusion into the study until hospitalization with or without AKI. To compare eGFR trajectories of individuals before hospitalization with AKI (cases) and without AKI (controls) we performed a nested case-control study with age at admission, sex, and length between study visit and hospitalization as matching criteria. The following variables were included in the model: age, log-transformed UACR (continuous), diabetes mellitus, arterial hypertension, congestive heart failure, peripheral artery disease, myocardial infarction, stroke, atrial fibrillation, BMI, smoking, polymedication (≥ 5 medication), number of prior hospitalizations, and log-transformed CRP (continuous) eGFR_BIS2: estimated glomerular filtration rate based on the creatinine and cystatin C-based BIS2 Eq. (15). BIS, Berlin Initiative Study. CI, confidence interval. AKI acute kidney injury. BMI, body mass index

Based on individual mean eGFR values, eGFR trajectories were modeled represented by the spaghetti plot-tableaus of individual raw data as well as the average eGFR trajectory for men and women (Fig. 3). The eGFR trajectories of men and women who developed hospital-acquired AKI showed a steeper decline prior to hospitalization compared to those without AKI. In the adjusted model, mean modeled eGFR in men with AKI was 4.2 and in women with AKI 7.4 ml/min/1.73 m² lower compared to their respective controls five years prior to hospitalization (Table S2). This difference increased to 7.5 in men and 10.2 ml/min/1.73 m² in women, modeled at 1 week prior to hospitalization. More details of model-based estimators of mean eGFR prior to hospitalization are shown in Table S2. We found similar results for the two models where only age or a lot more variables were included (Table S3, Figure S3A. and B.) and when using single biomarker creatinine or cystatin C-based eGFR (Figure S4A. and B.).

## Discussion

In our study of community-dwelling adults aged 70 + we found that 19% developed a first incident AKI over a median follow-up time of 8.8 years confirming that AKI is a very common in-hospital complication at old age [11, 22, 23]. In men, the IR of AKI was considerably higher compared to women. Age-stratified analysis revealed a steeply increasing IR with age reaching as high as 54.6/1000 py in nonagenarians. The BIS population is comparable with the older German general population in terms of important morbidities [24, 25]. Cardiovascular comorbidities had an important impact as the IR of AKI was more than twice as high in patients with pre-existing PAD, AF, reduced KF and CHF compared to those without the comorbidity. Applying a nested case-control design we found an increased rate of mean eGFR decline in patients before hospitalization with AKI compared to the non-AKI controls. The steeper decline of mean eGFR relative to the non-AKI group could be detected approximately five years prior to hospitalization. During the observation period 83% of study participants were hospitalized resulting in a very high number of older patients potentially at risk of developing hospital-acquired AKI.

Our IR are in line with findings from Hsu et al. who investigated IR of AKI in members of an integrated US health care delivery system (Kaiser Permanente) [26]. Overall IR of AKI was 18.1 and 35.45/1000 py among non-dialysis patients aged 70–79 and ≥ 80 years, respectively, comparable to our results. However, the very high IR of 54.6/1000 py we found in nonagenarians was striking and may indicate that the AKI risk accelerates quite dramatically with increasing age. Of note, the US analysis did not exclusively investigate hospital-acquired AKI but included all registered AKI cases. A Chinese study investigating in-hospital AKI in patients aged ≥ 60 reported rates as high as 27.8% for community and hospital-acquired AKI [23]. Since only hospitalized elderly patients were included, the authors did not report the IR of AKI for the general population.

Our findings suggest that hospital-acquired AKI risk in older adults is even higher than previously reported. The steep increase of IR by age may be explained by the fact that CV comorbidities are more prevalent in old age further aggravating AKI risk [27]. The fact that the IR of AKI is particularly high in patients with PAD, AF, reduced KF and CHF also suggests a strong cardio renal axis as underlying pathophysiological explanation for an increased susceptibility of AKI at old age [24]. Additionally, we know that GFR decreases with age [28, 29] consequently leading to an increasing risk of AKI.

In contrast to the claims data-based study from the US, participants of the BIS were seen biennially with repetitively assessed kidney function and albuminuria. We found that individuals who developed AKI had a baseline eGFR that was on average 9 ml/min/1.73 m² lower compared to the non-AKI group. Prevalence of albuminuria was nearly twice as high in the AKI compared to the non-AKI group highlighting the importance of kidney function and proteinuria monitoring pre-admission and confirming proteinuria as an important risk factor for AKI [30, 31] in old age. Medical treatment of common comorbidities further contributes to the AKI risk by potentially nephrotoxic effects of prescribed drugs.

We used a mixed-effects model to investigate differences of pre-admission eGFR trajectories based on multiple standardized creatinine and cystatin C measurements in patients with and without AKI. In contrast to our approach, the majority of studies investigating kidney function decline in the context of AKI focuses on worsening or recovery of eGFR post AKI [32–34]. Others included patient populations considerably sicker than ours investigating eGFR decline post-AKI leading up to initiation of dialysis [16, 35, 36]. In contrast to our prospective study approach, other studies calculated eGFR slope differences before and after AKI based on patient databases that include laboratory data and patients’ characteristics retrospectively, missing information for time-dependent confounder adjustment [37, 38]. This Danish study based on laboratory data found a drop in eGFR level and a declining eGFR slope following first-time AKI compared to the period before AKI [38].

Our findings suggest that long-term monitoring of kidney measures from medical records could improve identification of individuals at increased risk for AKI before hospital admission. Recent advances in digitalization of healthcare information such as electronic patient charts linked to laboratory data may take on a key role by providing immediate resumés of a patient’s health data including kidney function change over time. eGFR trajectories could complement automated pre-hospital risk assessment tools such as computer-based “AKI alerts” that have been established for detecting patients at risk for AKI to improve standards of care and proactively target patients at increased risk for adverse outcomes or death [39, 40]. Strategies for continuous prediction of future AKI based on deep learning approaches using electronic health record datasets [41] may increase their clinical utility by including personalized data on eGFR trajectories. However, further research including risk prediction models is necessary to evaluate whether eGFR trajectories may improve risk stratification to prevent AKI in older adults and how this information could be provided to healthcare professionals in an automated and comprehensible form.

The strengths of our study are its prospective design, a population-based setting, complementary individual insurance claims data and a fairly large cohort of old and very old adults with repeat eGFR values over more than eight years filling the data gap of IR of AKI and eGFR trajectories before hospitalization in a vulnerable population. In addition, eGFR values were measured including the endogenous biomarker cystatin C that may be more adequate in old age compared to creatinine, which is known to be confounded by sarcopenia [42].

Our study has also limitations. First, the AKI diagnosis was based on ICD-10 codes without information on AKI severity. However, we performed an extensive medical chart review of available hospital discharge reports to verify the AKI definition and were able to confirm all AKI cases for which we had medical charts. In 56% of medical reports we had access to laboratory results that reconfirmed the AKI diagnosis with serum creatinine values increasing our confidence in the validity of the claims data-based definition of hospital-acquired AKI. Second, as we used claims data on hospitalization with and without AKI we were missing information on physical status, laboratory results and urine output on admission and during the course of the AKI episode. Not surprisingly, in the nested case-control study median time of hospitalization was less than half in the non-AKI compared to the AKI group most probably due to a higher IR of in-hospital complications in patients with AKI. Finally, we chose a nested case-control approach selecting patients hospitalized with AKI and compared them to patients without AKI potentially loosing information by excluding individuals who did not fulfil the matching criteria. However, for only 20 cases we could not identify matching partners, therefore we estimate the loss of information as small.

## Conclusion

In conclusion, we found that AKI is a frequent in-hospital complication in individuals aged 70+ showing a strikingly rising IR with age. Compared to older patients hospitalized without AKI, older patients with AKI show a steeper eGFR decline already years prior to hospitalization independent of CV comorbidities. Hence, kidney function trajectories may improve risk stratification of older patients pre-admission.

## Electronic supplementary material

Below is the link to the electronic supplementary material.

Supplementary Material 1

## Data Availability

The dataset used and analysed during the current study is available from the corresponding author on reasonable request.

## List of Abbreviations

AF: Atrial Fibrillation
AKI: Acute kidney injury
AOK: Allgemeine Ortskrankenkasse
ATC: Anatomical Therapeutic Chemical (Classification System)
BIS: Berlin Initiative Study
BMI: Body Mass Index
CHF: Congestive Heart Failure
CI: Confidence Interval
CKD: Chronic kidney disease
CV: Cardiovascular
GFR: Glomerular filtration rate
eGFR: Estimated Glomerular filtration rate
ICD: International Classification of Diseases
IR: Incidence rate
IQR: Interquartile Range
KDIGO: Kidney Disease Improving Global Outcomes
MI: Myocardial infarction
PAD: Peripheral Artery Disease
PY: Patient Years
SD: Standard Deviation
UACR: Urinary Albumin Creatinine Ratio
