# Supplementary material for: Incidence of hospital-acquired acute kidney injury and trajectories of glomerular filtration rate in older adults
Source: BMC Nephrol. 2023 Aug 1;24:226. doi: 10.1186/s12882-023-03272-5 (PMC10394866; doi:10.1186/s12882-023-03272-5)
Supplement: Supplementary file 1 — Supplementary Material 1 [file 12882_2023_3272_MOESM1_ESM.docx]

**Supplemental Material**

| **Table of Contents** | **Page** |
| --- | --- |
| **Supplemental Methods** | **2** |
| **Table S1.** Baseline characteristics of whole study population including the participants who were never hospitalized | **4** |
| **Table S2.** Estimated mean eGFR in ml/min/1.73m² for men and women with and without AKI per time period of eGFR measurement pre hospitalization | **6** |
| **Figure S 1.** Flow chart | **7** |
| **Figure S 2.** Chart review: Verification of the claims data based AKI diagnosis (ICD-10: N17.X) | **8** |
| **Figures S3 A. and B.** eGFR trajectories with 95%CI (grey area) of individuals with AKI (green) and without AKI (red) for men and women calculated with two additional linear mixed-models in a nested case-control study (n = 404 men and 322 women). | **9** |
| **Figures S4 A. and B.** eGFR trajectories with 95%CI (grey area) of individuals with AKI (green) and without AKI (red) for men and women calculated with the combined creatinine and cystatin C eGFR (BIS2), the creatinine-based eGFR (EKFC_CREA) and the cystatin C-based eGFR (EKFC_CYS) | **11** |
| **STROBE Statement-checklist** | **12** |
| **References** | **14** |

**Supplemental Methods**

*Inclusion and Exclusion criteria of the Berlin Initiative Study (1)*

Inclusion criteria:

- *Membership in the “AOK Nordost - Die Gesundheitskasse” health insurance*
- *Residency in Berlin*
- *Age ≥70 years*

Exclusion criteria:

- *Renal replacement therapy or kidney transplantation*
- *Severe care dependency*

*Covariates*

The following covariates were derived from BIS data: age, sex, number of medications, smoking, physical exercise, blood pressure (mmHg), body mass index (BMI, kg/m²), diabetes mellitus (antidiabetic drug intake (ATC-based) or HbA1clevel ≥6.5%), arterial hypertension (treatment with at least one antihypertensive medication (ATC-based), except for monotherapy with a loop diuretic), and peripheral artery disease (PAD) (angiography, dilatation/stenting or amputation due to peripheral vascular disease). We calculated estimated glomerular filtration rate (eGFR) with the BIS2 equation based on creatinine and cystatin C (2) categorized in ≥60 and <60 ml/min per 1.73 m² (reduced kidney function). Urinary albumin-to-creatinine ratio (UACR) was categorized in albuminuria (≥30 mg/g in spot urine analysis) and no albuminuria (<30 mg/g). Serum creatinine and cystatin C were measured with standardized laboratory methods. Further details can be found elsewhere (2). The definition of the following comorbidities include self-reported and/or claims data based on the International Classification of Diseases Tenth version coding (ICD-10): myocardial infarction (self-reported or ICD-10: I21, I22, I25.2), atrial fibrillation (AF) (at least one inpatient or two outpatient ICD-10: I48.x), stroke (self-reported or ICD-10: G45.x, I61.x, I62.x, I63.x, I64.x), congestive heart failure (CHF) (at least one inpatient or two outpatient ICD-10: I11.0, I13.0, I13.2, I50.x), cancer (ICD-10: C00.x-C97.x, except C44.x). Kidney replacement therapy was defined based on claims data including the in-hospital ICD-10 diagnosis Z49.1 or Z49.2 in combination with the OPS code 8-853, 8-854 or 8-855.

**Table S1.** Baseline characteristics of whole study population including the participants who were never hospitalized

|  | **Total n=2020 (%)** | **AKI**  **n=383 (%)** | **Without AKI**  **n=1518 (%)** | **Non-hospital n=119** |
| --- | --- | --- | --- | --- |
| Age, years (mean, ± SD) | 80.4 ± 6.7 | 82.2 ± 6.3 | 80.1 ± 6.6 | 77.4 ± 6.5 |
| 70-74 | 557 (27.6) | 61 (15.9) | 436 (28.7) | 60 (50.4) |
| 75-79 | 470 (23.3) | 78 (20.4) | 369 (24.3) | 23 (19.3) |
| 80-84 | 421 (20.8) | 103 (26.9) | 300 (19.8) | 18 (15.1) |
| 85-89 | 375 (18.6) | 95 (24.8) | 269 (17.7) | 11 (9.2) |
| ≥90 | 197 (9.8) | 46 (12.0) | 144 (9.5) | 7 (5.9) |
| Female | 1068 (52.9) | 170 (44.4) | 834 (54.9) | 64 (53.8) |
| Number of medications | 4.8 ± 3.0 | 6.1 ± 3.1 | 4.6 ± 2.9 | 3.1 ± 2.3 |
| Smoking |  |  |  |  |
| never | 1022 (50.6) | 169 (44.1) | 793 (52.2) | 60 (50.4) |
| past | 888 (44.0) | 194 (50.7) | 642 (42.3) | 52 (43.7) |
| current | 108 (5.3) | 20 (5.2) | 81 (5.3) | 7 (5.9) |
| Physical activity (>30 min) |  |  |  |  |
| <1x /week | 509 (25.2) | 124 (32.4) | 362 (23.8) | 23 (19.3) |
| 1-5x /week | 939 (46.5) | 157 (41.0) | 723 (47.6) | 59 (49.6) |
| >5x /week | 567 (28.1) | 100 (26.1) | 430 (28.3) | 37 (31.1) |
| Systolic blood pressure, mmHg | 145.5 ± 21.8 | 146.4 ± 22.4 | 145.2 ± 21.8 | 146.0 ± 20.1 |
| Diastolic blood pressure, mmHg | 81.3 ± 13.0 | 80.1 ± 13.4 | 81.6 ± 13.0 | 81.8 ±11.7 |
| BMI, kg/m² | 27.7 ± 4.2 | 28.4 ± 4.5 | 27.6 ± 4.2 | 27.2 ± 3.9 |
| BMI <30, kg/m² | 1489 (73.6) | 257 (67.1) | 1137 (74.9) | 92 (77.3) |
| BMI ≥30, kg/m² | 533 (26.4) | 125 (32.6) | 381 (25.1) | 27 (22.7) |
| Creatinine, mg/dl | 1.0 ± 0.3 | 1.2 ± 0.4 | 1.0 ± 0.3 | 0.9 ± 0.3 |
| Cystatin C, mg/l | 1.1 ± 0.4 | 1.3 ± 0.4 | 1.1 ± 0.3 | 1.0 ± 0.3 |
| Albumin, g/l | 40.0 ± 3.1 | 39.5 ± 3.1 | 40.0 ± 3.0 | 40.8 ± 2.9 |
| CRP, mg/l | 1.8 [0.9; 3.6] | 2.4 [1.2; 4.8] | 1.7 [0.9; 3.3] | 1.4 [0.9; 3.0] |
| HbA1c, % | 6.0 [5.8; 6.4] | 6.2 [5.8; 6.5] | 6.0 [5.8; 6.4] | 6.0 [5.7; 6.4] |
| Urea, mg/dl | 41.6 [34.4; 51.2] | 47.2 [38.8; 59.4] | 40.4 [33.9; 49.6] | 38.9 [32.6; 48.8] |
| eGFRBIS2, ml/min/1.73m² | 58.4 ± 15.1 | 50.5 ± 14.4 | 59.8 ± 14.6 | 64.9 ± 14.0 |
| eGFRBIS2 ≥60 ml/min/1.73m² | 956 (47.3) | 95 (24.8) | 783 (51.6) | 78 (65.5) |
| eGFRBIS2 <60 ml/min/1.73m² | 1063 (52.6) | 288 (75.2) | 734 (48.4) | 41 (34.5) |
| UACR, mg/g | 10.6 [4.5; 30.0] | 17.6 [6.6; 71.6] | 9.6 [4.3; 27.0] | 6.0 [3.6; 14.6] |
| UACR <30 mg/g | 1502 (74.4) | 229 (59.8) | 1169 (77.0) | 104 (87.4) |
| UACR ≥30 mg/g | 501 (24.8) | 151 (39.4) | 336 (22.1) | 14 (11.8) |
| Diabetes mellitus | 522 (25.8) | 138 (36.0) | 362 (23.8) | 22 (18.5) |
| Arterial hypertension | 1595 (79.0) | 342 (89.3) | 1178 (77.6) | 75 (63.0) |
| Congestive heart failure | 515 (25.5) | 136 (35.5) | 365 (24.0) | 14 (11.8) |
| Atrial fibrillation | 293 (14.5) | 85 (22.2) | 201 (13.2) | 7 (5.9) |
| Peripheral artery disease | 122 (6.0) | 39 (10.2) | 80 (5.3) | 3 (2.5) |
| Stroke | 216 (10.7) | 55 (14.4) | 154 (10.1) | 7 (5.9) |
| Myocardial infarction | 298 (14.8) | 92 (24.0) | 195 (12.8) | 11 (9.2) |
| Anemia^a^, n (%) | 344 (17.0) | 94 (24.5) | 237 (15.6) | 13 (10.9) |
| Cancer | 553 (27.3) | 112 (29.2) | 412 (27.1) | 28 (23.5) |

**Legend:** Values for continuous variables given as mean ± standard deviation, or median [interquartile range]; for categorical variables, as number (percentage). ^a^Anemia: haemoglobin in men <13 g/dl and in women <12 mg/dl. Missing values for anthropometric or laboratory values do not exceed 3%.

Abbreviations: BIS, Berlin Initiative Study; eGFR, estimated glomerular filtration rate by creatinine- and cystatin C–based BIS2 equation; BMI, body mass index; CRP, C-reactive protein; UACR, urinary albumin-creatinine ratio; HbA1c, glycosylated haemoglobin type A1C.

**Table S2.** Model based estimators of mean eGFR for men and women with and without AKI “5 years “and “1 week” prior to hospitalization.

| **Model based estimators of (mean) eGFR for patients with and without AKI** | **5 years prior to hospitalization** | | **1 week prior to hospitalization** | |
| --- | --- | --- | --- | --- |
|  | **men**  **(n = 404)** | **women**  **(n=322)** | **men**  **(n = 404)** | **women**  **(n=322)** |
| **Adjusted model** |  |  |  |  |
| AKI; mean eGFR in ml/min/1.73m² (CI) | 51.5  (49.6-53.3) | 48.4  (46.6-50.3) | 45.8  (43.2-48.4) | 41.9  (39.4-44.5) |
| Non AKI; mean eGFR in ml/min/1.73m² (CI) | 55.7  (53.8-57.6) | 55.8  (53.8-57.7) | 53.3  (51.2-55.5) | 52.1  (49.7-54.5) |

**Legend:** The model was performed to investigate eGFR trajectories including time, AKI and the interaction of both and the following variables: age, log-transformed UACR (continuous), diabetes mellitus, arterial hypertension, CHF, PAD, myocardial infarction, stroke, AF, BMI, smoking, polymedication (≥5 medications), number of prior hospitalizations, and log-transformed CRP (continuous). eGFR trajectories were modeled using time, AKI and the interaction of both. Abbreviations: eGFR_BIS2, estimated glomerular filtration rate based on the creatinine and cystatin C-based BIS2 equation (2). BIS, Berlin Initiative Study. AKI, acute kidney injury. CI, 95% confidence interval.

**Table S3.** Model based estimators of mean eGFR in for men and women with and without AKI “5 years “and “1 week” prior to hospitalization.

| **Model based estimators of (mean) eGFR for patients with and without AKI** | **5 years prior to hospitalization** | | **1 week prior to hospitalization** | |
| --- | --- | --- | --- | --- |
|  | **men**  **(n = 404)** | **women**  **(n=322)** | **men**  **(n = 404)** | **women**  **(n=322)** |
| **A. First additional model** |  |  |  |  |
| AKI; mean eGFR in ml/min/1.73m² (CI) | 51.0  (49.1-52.9) | 48.2  (46.3-50.1) | 43.3  (40.6-46.0) | 40.2  (37.7-42.8) |
| Non AKI; mean eGFR in ml/min/1.73m²(CI) | 57.4  (55.4-59.3) | 57.0  (55.0-59.0) | 53.1  (50.8-55.4) | 52.4  (49.9-54.9) |
| **B. Second additional model** |  |  |  |  |
| AKI; mean eGFR in ml/min/1.73m² (CI) | 51.2  (49.3-53.1) | 48.3  (46.4-50.3) | 45.0  (42.3-47.7) | 41.1  (38.5-43.7) |
| Non AKI; mean eGFR in ml/min/1.73m² (CI) | 56.3  (54.3-58.3) | 56.4  (54.4-58.4) | 53.0  (50.8-55.3) | 52.1  (49.6-54.5) |

**Legend:** Two additional models (A, B) were performed to investigate eGFR trajectories including time, AKI and the interaction of both. The first model (A) included only age and the second model (B) included CV comorbidities in addition to model (A): log-transformed UACR (continuous), diabetes mellitus, arterial hypertension, CHF, PAD, myocardial infarction, stroke, and AF. Abbreviations: eGFR_BIS2, estimated glomerular filtration rate based on the creatinine and cystatin C-based BIS2 equation (2). BIS, Berlin Initiative Study. AKI, acute kidney injury. CI, 95% confidence interval.

**Figure S1.** Flow chart

**Figure S2.** Chart review: Verification of the claims data based AKI diagnosis (ICD-10: N17.X)


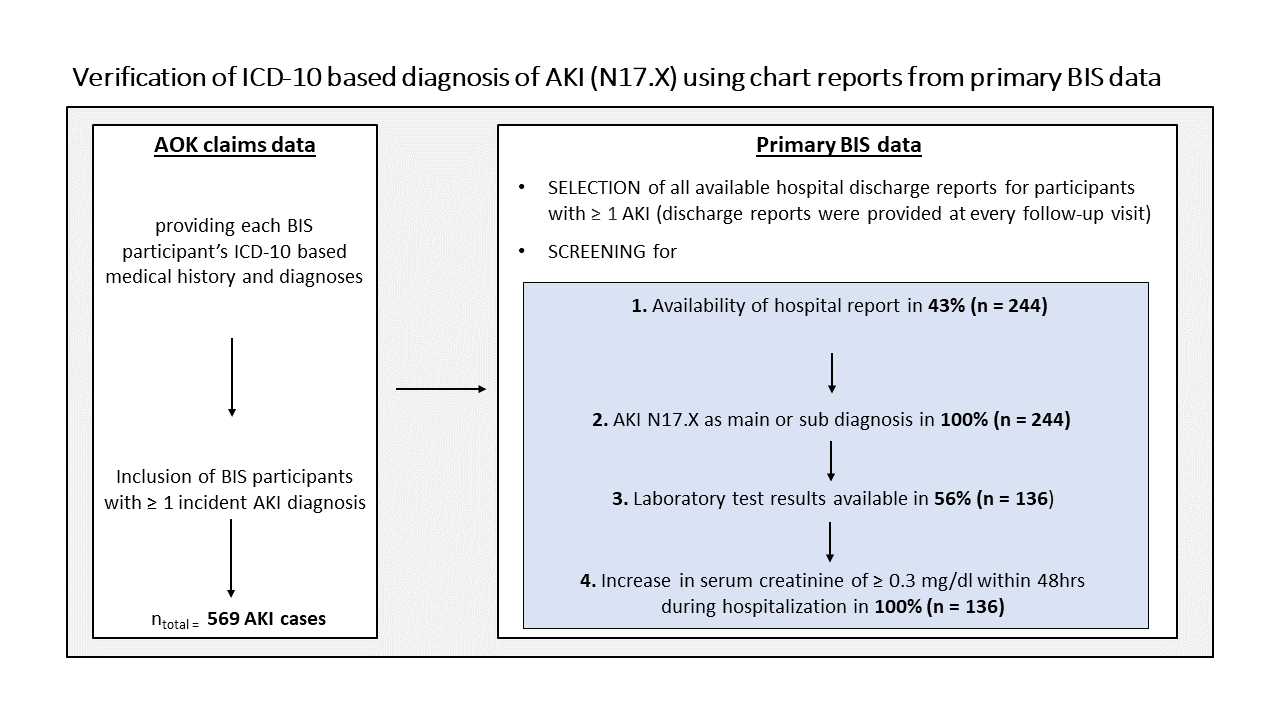


**Figures S3 A. and B.** eGFR trajectories with 95%CI (grey area) of individuals with AKI (green) and without AKI (red) for men and women calculated with two additional linear mixed-models in a nested case-control study


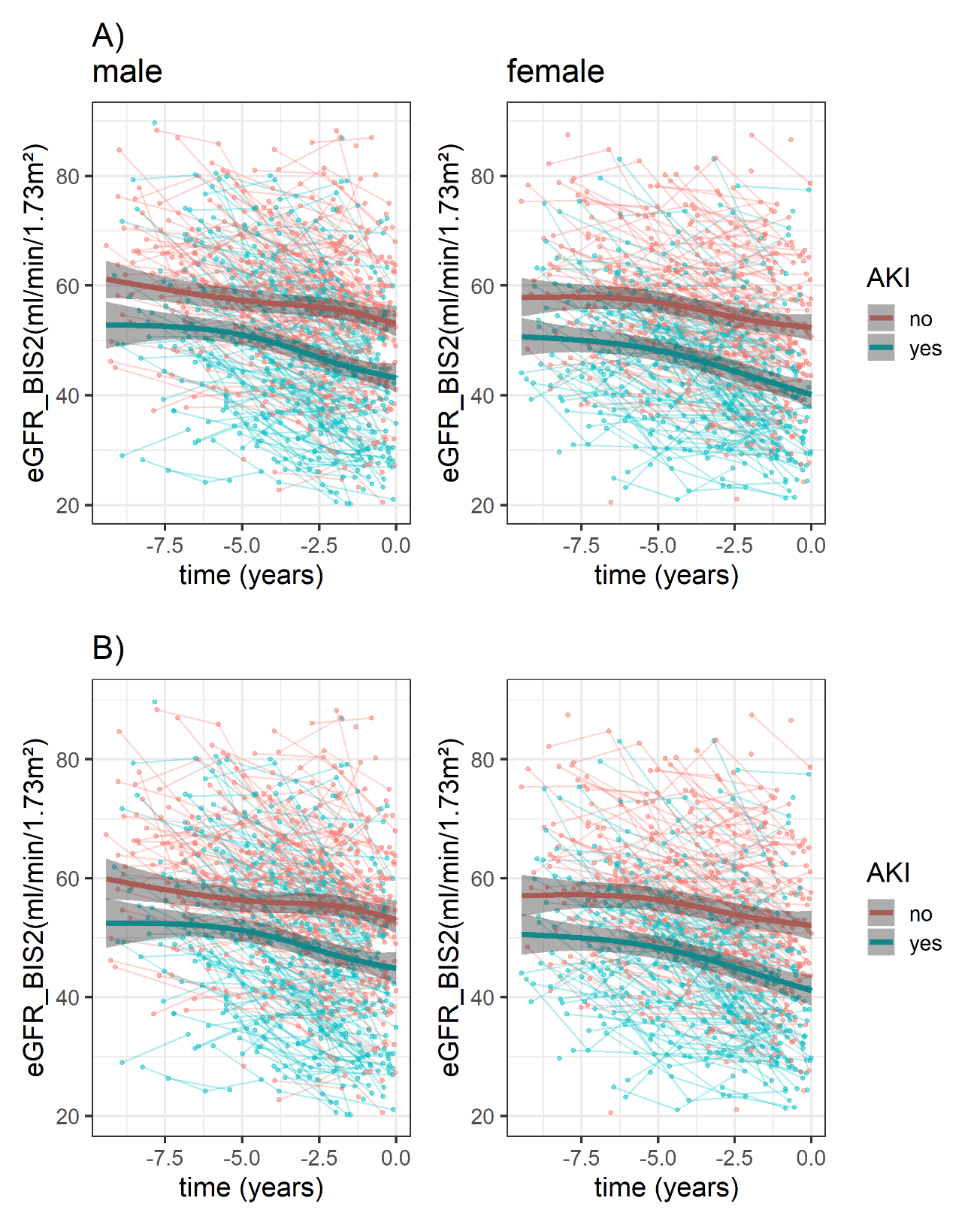


The x-axes shows the time (years) from inclusion into the study until hospitalization with or without AKI. A. shows the mixed-effects model including only age as linear and quadratic term. B. shows a second mixed-effects model including age, log-transformed UACR (continuous), diabetes mellitus, arterial hypertension, congestive heart failure, peripheral artery disease, myocardial infarction, stroke, and atrial fibrillation. eGFR_BIS2: estimated glomerular filtration rate based on the creatinine and cystatin C-based BIS2 equation (2). BIS, Berlin Initiative Study. CI, confidence interval. AKI acute kidney injury. BMI, body mass index.

**Figures S4 A. and B.** eGFR trajectories with 95%CI (grey area) of individuals with AKI (green) and without AKI (red) for men and women calculated with the combined creatinine and cystatin C eGFR (BIS2), the creatinine-based eGFR (EKFC_CREA) and the cystatin C-based eGFR (EKFC_CYS)


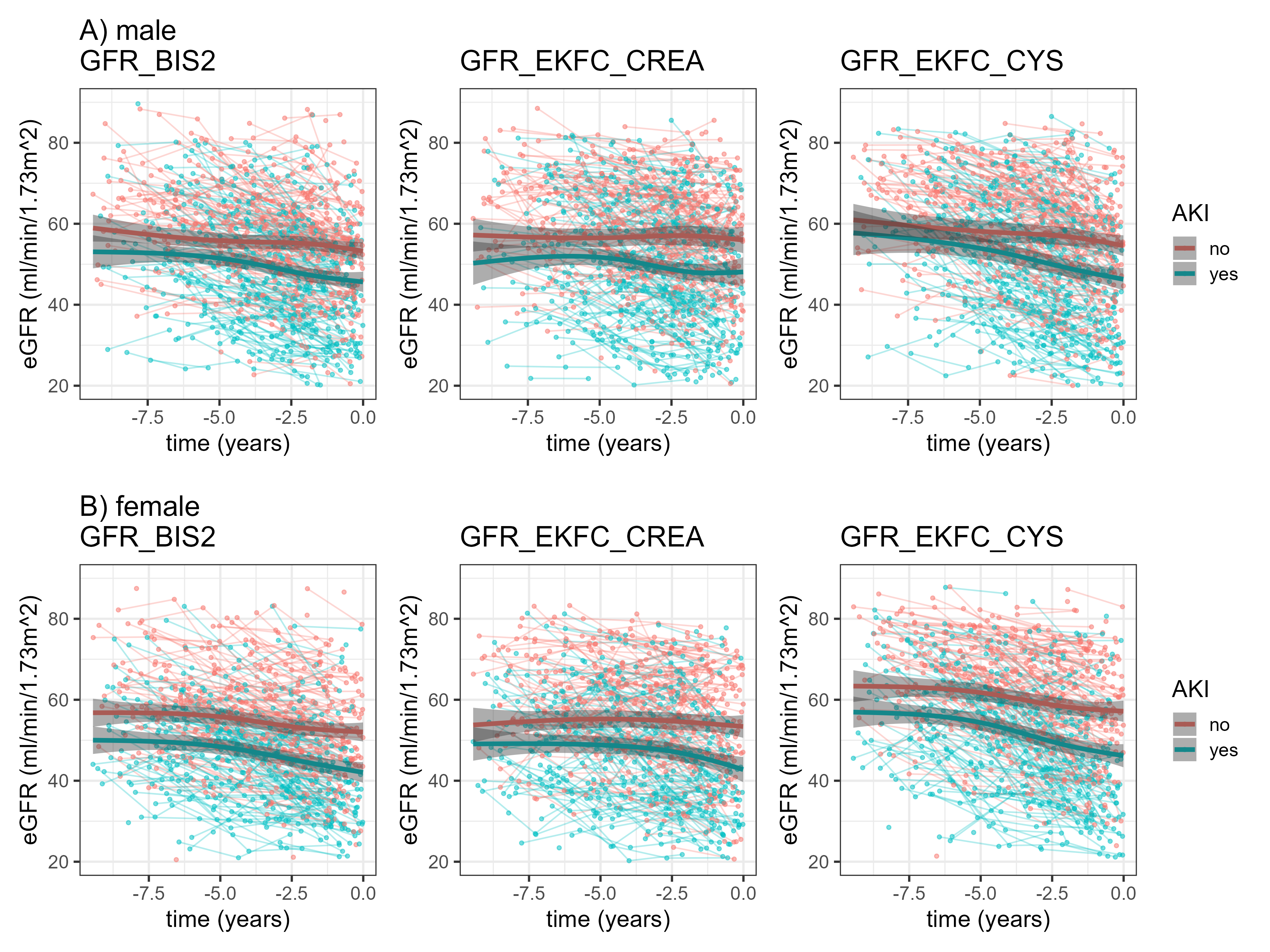


eGFR trajectories were estimated with a mixed-effect model based on pooled results of multiple imputed data with 95% confidence intervals (grey area) for men with (n = 202) and without (n = 202) AKI and women with (n =161) and without (n = 161) AKI .The x-axis shows the time (years) from inclusion into the study until hospitalization with or without AKI. To compare eGFR trajectories of individuals before hospitalization with AKI (cases) and without AKI (controls) we performed a nested case-control study with age at admission, sex, and length between study visit and hospitalization as matching criteria. The following variables were included in the model: age, log-transformed UACR (continuous), diabetes mellitus, arterial hypertension, congestive heart failure, peripheral artery disease, myocardial infarction, stroke, atrial fibrillation, BMI, smoking, polymedication (≥5 medication),number of prior hospitalizations, and log-transformed CRP (continuous).

GFR_BIS2: estimated glomerular filtration rate based on the creatinine and cystatin C-based BIS2 equation (2), GFR_EKFC_CREA: the creatinine-based EKFC equation (3)and GFR_EKFC_CYS: the cystatin C-based EKFC equation(4). BIS, Berlin Initiative Study. EKFC, European Kidney Function Consortium. CI, confidence interval. AKI acute kidney injury. BMI, body mass index.

**STROBE Statement**—Checklist of items that should be included in reports of ***cohort studies***

|  | **Item No** | **Recommendation** | **Page No** |
| --- | --- | --- | --- |
| **Title and abstract** | 1 | (*a*) Indicate the study’s design with a commonly used term in the title or the abstract | Abstract paragraph Methods |
|  |  | (*b*) Provide in the abstract an informative and balanced summary of what was done and what was found | Abstract |
| **Introduction** | | | |
| Background/rationale | 2 | Explain the scientific background and rationale for the investigation being reported | 3 |
| Objectives | 3 | State specific objectives, including any prespecified hypotheses | 3/4 |
| **Methods** | | | |
| Study design | 4 | Present key elements of study design early in the paper | 4 |
| Setting | 5 | Describe the setting, locations, and relevant dates, including periods of recruitment, exposure, follow-up, and data collection | 4/5 |
| Participants | 6 | (*a*) Give the eligibility criteria, and the sources and methods of selection of participants. Describe methods of follow-up | 4-6, Supp. |
|  |  | (*b*) For matched studies, give matching criteria and number of exposed and unexposed | 4-5 |
| Variables | 7 | Clearly define all outcomes, exposures, predictors, potential confounders, and effect modifiers. Give diagnostic criteria, if applicable | 5/6, Supp. |
| Data sources/ measurement | 8* | For each variable of interest, give sources of data and details of methods of assessment (measurement). Describe comparability of assessment methods if there is more than one group | 4-6 |
| Bias | 9 | Describe any efforts to address potential sources of bias | 4-7, Supp. |
| Study size | 10 | Explain how the study size was arrived at | 4-5, Supp. (Flow chart) |
| Quantitative variables | 11 | Explain how quantitative variables were handled in the analyses. If applicable, describe which groupings were chosen and why | 7 |
| Statistical methods | 12 | (*a*) Describe all statistical methods, including those used to control for confounding | 6-7 |
|  |  | (*b*) Describe any methods used to examine subgroups and interactions | 6-7 |
|  |  | (*c*) Explain how missing data were addressed | 7 |
|  |  | (*d*) If applicable, explain how loss to follow-up was addressed | N/A |
|  |  | (*e*) Describe any sensitivity analyses | N/A |

| **Results** | | | |  |
| --- | --- | --- | --- | --- |
| Participants | | 13* | (a) Report numbers of individuals at each stage of study—eg numbers potentially eligible, examined for eligibility, confirmed eligible, included in the study, completing follow-up, and analysed | 8/9, Supp. Flow chart |
|  |  |  | (b) Give reasons for non-participation at each stage | 8, Flow chart |
|  |  |  | (c) Consider use of a flow diagram | ✓ |
| Descriptive data | | 14* | (a) Give characteristics of study participants (eg demographic, clinical, social) and information on exposures and potential confounders | 8/9 |
|  |  |  | (b) Indicate number of participants with missing data for each variable of interest | ✓ |
|  |  |  | (c) Summarise follow-up time (eg, average and total amount) | ✓ |
| Outcome data | | 15* | Report numbers of outcome events or summary measures over time | ✓ |
| Main results | 16 | (*a*) Give unadjusted estimates and, if applicable, confounder-adjusted estimates and their precision (eg, 95% confidence interval). Make clear which confounders were adjusted for and why they were included | | 9/10 Supp. |
|  |  | (*b*) Report category boundaries when continuous variables were categorized | | ✓ |
|  |  | (*c*) If relevant, consider translating estimates of relative risk into absolute risk for a meaningful time period | | N/A |
| Other analyses | 17 | Report other analyses done—eg analyses of subgroups and interactions, and sensitivity analyses | | 8-10 |
| **Discussion** | | | | |
| Key results | 18 | Summarise key results with reference to study objectives | | 10/11 |
| Limitations | 19 | Discuss limitations of the study, taking into account sources of potential bias or imprecision. Discuss both direction and magnitude of any potential bias | | 13/14 |
| Interpretation | 20 | Give a cautious overall interpretation of results considering objectives, limitations, multiplicity of analyses, results from similar studies, and other relevant evidence | | ✓ |
| Generalisability | 21 | Discuss the generalisability (external validity) of the study results | | 11-13 |
| **Other information** | | | | |
| Funding | 22 | Give the source of funding and the role of the funders for the present study and, if applicable, for the original study on which the present article is based | | ✓ |

*Give information separately for exposed and unexposed groups.

**Note:** An Explanation and Elaboration article discusses each checklist item and gives methodological background and published examples of transparent reporting. The STROBE checklist is best used in conjunction with this article (freely available on the Web sites of PLoS Medicine at http://www.plosmedicine.org/, Annals of Internal Medicine at http://www.annals.org/, and Epidemiology at http://www.epidem.com/). Information on the STROBE Initiative is available at http://www.strobe-statement.org.

1. Schaeffner ES, van der Giet M, Gaedeke J, Tolle M, Ebert N, Kuhlmann MK, et al. The Berlin initiative study: the methodology of exploring kidney function in the elderly by combining a longitudinal and cross-sectional approach. Eur J Epidemiol. 2010;25(3):203-10.

2. Schaeffner ES, Ebert N, Delanaye P, Frei U, Gaedeke J, Jakob O, et al. Two novel equations to estimate kidney function in persons aged 70 years or older. Ann Intern Med. 2012;157(7):471-81.

3. Pottel H, Bjork J, Courbebaisse M, Couzi L, Ebert N, Eriksen BO, et al. Development and Validation of a Modified Full Age Spectrum Creatinine-Based Equation to Estimate Glomerular Filtration Rate : A Cross-sectional Analysis of Pooled Data. Ann Intern Med. 2021;174(2):183-91.

4. Pottel H, Bjork J, Rule AD, Ebert N, Eriksen BO, Dubourg L, et al. Cystatin C-Based Equation to Estimate GFR without the Inclusion of Race and Sex. N Engl J Med. 2023;388(4):333-43.
